# *In Silico* Identification of Specialized Secretory-Organelle Proteins in Apicomplexan Parasites and *In Vivo* Validation in *Toxoplasma gondii*

**DOI:** 10.1371/journal.pone.0003611

**Published:** 2008-10-31

**Authors:** ZhongQiang Chen, Omar S. Harb, David S. Roos

**Affiliations:** Department of Biology, Penn Genomic Frontiers Institute, and the Graduate Program in Genomics and Computational Biology, University of Pennsylvania, Philadelphia, Pennsylvania, United States of America; New England Biolabs, United States of America

## Abstract

Apicomplexan parasites, including the human pathogens *Toxoplasma gondii* and *Plasmodium falciparum*, employ specialized secretory organelles (micronemes, rhoptries, dense granules) to invade and survive within host cells. Because molecules secreted from these organelles function at the host/parasite interface, their identification is important for understanding invasion mechanisms, and central to the development of therapeutic strategies. Using a computational approach based on predicted functional domains, we have identified more than 600 candidate secretory organelle proteins in twelve apicomplexan parasites. Expression in transgenic *T. gondii* of eight proteins identified *in silico* confirms that all enter into the secretory pathway, and seven target to apical organelles associated with invasion. An *in silico* approach intended to identify possible host interacting proteins yields a dataset enriched in secretory/transmembrane proteins, including most of the antigens known to be engaged by apicomplexan parasites during infection. These domain pattern and projected interactome approaches significantly expand the repertoire of proteins that may be involved in host parasite interactions.

## Introduction

The phylum apicomplexa encompasses more than 5000 unicellular eukaryotic parasite species [1], including important human pathogens such as *Toxoplasma gondii* (responsible for toxoplasmosis) [2] and *Plasmodium spp.* (which cause malaria) [3]. As obligate intracellular parasites, all apicomplexans must invade and establish a parasitophorous vacuole (PV) within their respective host cells in order to survive. Specialized secretory organelles known as micronemes, rhoptries and dense granules deliver cargo proteins in a coordinated fashion during the invasion process [4]. Small cigar-shaped micronemes and larger club-shaped rhoptries are located in the anterior part of the parasite and are thought to be involved in host cell invasion and establishment of the PV, respectively; spherical dense granules are more broadly distributed and are thought to be required for general secretion and PV maintenance [5], [6].

A wide variety of studies have sought to identify proteins associated with these specialized secretory organelles, including: biochemical characterization of subcellular fractions [7]–[12], computational analysis and tagging of candidate proteins [13]–[15], direct antibody staining [16], [17], site-directed mutagenesis to define targeting signals [18], and proteomic analysis of the secretome [19]–[21]. These methods have identified many candidates, although the catalog remains incomplete.

Proteins trafficking to parasite secretory compartments typically possess a classical N-terminal signal sequence. In addition, secondary targeting signals are responsible for localization to the apical secretory organelles [18], [22]–[25], although these motifs are insufficiently defined, or insufficiently specific, to allow genome-wide identification of microneme and rhoptry proteins. Many microneme proteins (MICs) also contain well-conserved functional domains associated with adhesive or protease activity [26], [27]. Such domains are widely distributed, among multiple protein classes. For example, the thrombospondin type 1 (TSP-1), von Willebrand Factor A (VWA) and plasminogen apple nematode (PAN) domains, originally defined based on their role in mediating protein-protein and cell-cell interactions in mammalian cells [28]–[32], are also prevalent in parasite microneme proteins, where they are thought to interact with the extracellular milieu to mediate motility, attachment and/or invasion into host cells [26], [33], [34]. For example, the TSP-1 domain of *Pf*TRAP is essential for interaction with the sulfated glycoconjugates of hepatocytes, and the PAN domain of *Tg*MIC4 is crucial for host-cell binding [35], [36]. Protease domains are indispensable for the maturation and activation of microneme proteins [37]–[39]. The conservation of these functional domains makes it possible to exploit computational approaches to detect candidate microneme proteins encoded by apicomplexan parasite genomes. With the emergence of large-scale human interactome datasets, we can also contemplate the identification of candidate host interacting partners.

In order to identify new proteins likely to be associated with host cell invasion by apicomplexan parasites, we have developed an integrated computational approach for mining currently available apicomplexan genomes. As a first step, a list of all Pfam domains present in known apicomplexan microneme proteins was used to define signatures, which were employed to search the completed genome sequences for twelve parasite species (*T. gondii*, *Babesia bovis*, two species of *Cryptosporidium*, two species of *Theileria*, and six species of *Plasmodium*). The resulting set of predicted proteins is highly enriched in N-terminal signal peptide (SP) or signal anchor (SA) predictions, and testing of eight candidates by transfection into *T. gondii* suggests that many are targeted to the apical organelles. An *in silico* approach was also employed to mine available human interactome datasets for proteins that might engage with parasite adhesive domains. In aggregate, this study provides a catalog of candidate parasite and host proteins that may play roles in invasion and/or intracellular survival of apicomplexan parasites.

## Materials and Methods

### Computational approaches for domain discovery and sequence analysis

**Figure 1:**
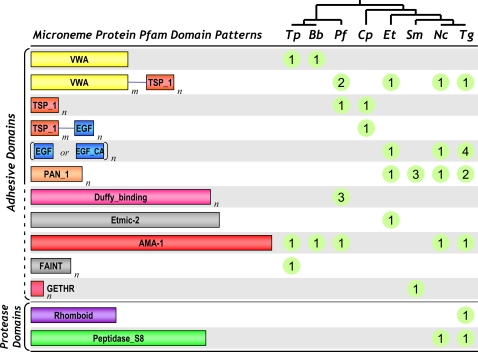
Pfam domains and domain patterns in the microneme proteins of apicomplexan parasites. Microneme proteins are shown for representative species in each parasite genus: *Tp*, *Theileria parva*; *Bb*, *Babesia bovis*; *Pf*, *Plasmodium falciparum*; *Cp*, *Cryptosporidium parvum*; *Et*, *Eimeria tenella*; *Sm*, *Sarcocystis muris*; *Nc*, *Neospora caninum*; *Tg*, *Toxoplasma gondii*. The tree provided at top indicates phylogenetic relationships (branch lengths not proportional to phylogenetic distance). Known microneme proteins are indicated by green circles, and Pfam domains by colored boxes (lengths proportional to the actual domain length, except for AMA-1). Subscripts (*m*,*n*) mark domains that may be repeated. Domains include: VWA, von Willebrand factor type A domain; TSP_1, thrombospondin type 1 domain; EGF, epidermal growth factor-like domain; EGF-CA, calcium binding EGF domain; PAN_1, PAN domain; GETHR, GETHR pentapeptide repeat; FAINT, frequently associated in *Theileria*; AMA-1, apical membrane antigen 1; Etmic-2, microneme protein Etmic-2; Duffy_binding, Duffy binding domain; Rhomboid, rhomboid family; Peptidase_S8, subtilase family. Accession numbers (where available): *Tp*: VWA: EAN31658, FAINT: AAA18217, AMA-1: XP_766171; *Bb*, VWA: AAS58046; AMA-1: AAS58045; *Pf*: VWAmTSP_1n: CAD52497 AAC46961; TSP_1n: AAN36262; AMA-1: AAN35928; Duffy_binding: AAK49521 AAS46319 P19214; *Cp*: TSP_1n: AAC48311; TSP_1mEGFn: AAC16621; *Et*: VWAmTSP_1n: AAD03350; (EGF or EGF_CA)n: CAC34726; PAN_1n: CAB52368; Etmic-2: AAD05566; *Sm*: PAN_1n: AAF36512 Q08668 Q26539; GETHR: CAA81555; *Nc*: VWAmTSP_1n: AAF01565; (EGF or EGF_CA)n: AAF19184; PAN_1n: DAA05464; AMA-1: BAF45372; Peptidase_S8: AAF04257; *Tg*, VWAmTSP_1n: AAB63303; (EGF or EGF_CA)n: AAD28185 CAB56644 AAK35070 AAK19757; PAN_1n: CAJ20618 AAD33906; AMA-1: AAB65410;Peptidase_S8: AAK94670; Rhomboid: AAT29065. This figure does not include the following validated microneme proteins, which lack Pfam domains: *Pf*: AAM47192 AAN35478 CAD49152; *Cp*: AAC98153; *Et*: AAR87666 AAR87667; *Sm*: AAK35069; *Nc*: AAL37729 AAG32025 AAN16380 AAK74070; *Tg*: CAA96466 CAA70921 AAK19758 AAG32024 AAN16379 AAK58479 AAK51546.

To identify Pfam domains present in microneme proteins, we first compiled a list of all known microneme antigens from representative apicomplexan parasites, based on an exhaustive search of biological sequence and literature databases for proteins annotated with the keywords “microneme” or “micronemal”. In all cases, the primary literature citation was consulted for further verification. Two *P. falciparum* proteins (Genbank CAB37326, ABW16954) were excluded from the ‘known microneme protein’ dataset due to conflicting localization data [40]–[43], although this had no effect on final list of microneme domains, as rhomboid and peptidase_S8 domains are represented by other microneme proteins (e.g. AAK94670, AAT29065). This dataset was then searched for Pfam motifs (v21.0) using hmmpfam (http://hmmer.janelia.org/) with ‘gathering cutoff’ scores [44], to generate a comprehensive list of all domains and domain patterns represented. Remarkably, no Pfam domains were identified other than adhesin and protease domains, with the former dominant, as shown in Figure 1.

In order to identify parasite proteins containing the above domains, predicted proteins from all completely sequenced apicomplexan parasites (http://ApiDB.org) were first searched against Pfam as above. The results were then filtered based on the presence of any of the domains defined in Figure 1. Results from this analysis are provided in Table 1.

**Table 1:** Distribution of proteins containing one or more Pfam domains known to be found in microneme proteins, for twelve complete apicomplexan parasite genomes*.

*Adhesive Domain Patterns*	*Bb*	*Ch*	*Cp*	*Pb*	*Pc*	*Pk*	*Pf*	*Pv*	*Py*	*Ta*	*Tp*	*Tg*
AMA-1	**1**			**2**	**2**	**1**	**1**	**1**	**1**	**1**	**1**	**3**
VWA	**1**			**1**	**3**	**2**	**2**	**1**	**3**	**2**	**2**	**1**
VWA;TSP_1				**2**	**1**	**1**	**2**	**2**	**2**			**1**
TSP_1	**6**	**3**	**4**	**5**	**4**	**5**	**4**	**4**	**4**	**3**	**2**	**8**
TSP_1;EGF		**2**	**3**									
TSP_1;Kringle;TSP_1		**1**	**1**									
TSP_1;VWA					**1**			**1**				
Notch;TSP_1;Notch;Sushi												**1**
Notch;TSP_1;Notch			**1**									
zf-C3HC4;TSP_1											**1**	
EGF-like(EGF,EGF_CA)	**1**	**3**	**3**	**1**	**2**	**4**	**3**	**3**	**2**			**15**
EGF;PAN_1		**1**	**1**									
MSP1_C;EGF							**1**	**1**				
PAN_1		**3**	**3**									**14**
PAN_1;TSP_1												**1**
Duffy_binding				**1**	**1**	**2**	**18**	**1**	**1**			
Duffy_binding;Merozoite_SPAM							**2**					
Duffy_binding;PFEMP;Duffy_binding							**9**					
Duffy_binding;PFEMP							**45**					
Duffy_binding;PFEMP;Duffy_binding;Hep_Hag							**1**					
Duffy_binding;PFEMP;Hep_Hag							**5**					
GETHR		**1**	**1**			**3**	**1**	**10**	**3**			
GETHR;DEAD;Helicase_C;GETHR								**1**				
GETHR;Leu_Phe_trans									**1**			
GETHR;Myb_DNA-binding									**1**			
GETHR;MT-A70								**1**				
GETHR;Pentapeptide_2;GETHR								**2**				
GETHR;PseudoU_synth_2;GETHR								**1**				
GETHR;VPS9						**1**						
RRM_1;GETHR							**1**		**1**			
S1;GETHR								**1**				
FAINT										**92**	**116**	
FAINT;Abhydrolase_1											**1**	
FAINT;PT;FAINT											**1**	
FAINT;Tash_PEST										**8**	**8**	
FAINT;Tash_PEST;FAINT											**2**	
FAINT;AT_hook;Tash_PEST										**1**		
FAINT;SRR										**1**		
FAINT;Tash_PEST;AT_hook										**1**		
FAINT;Tash_PEST;AT_hook;FAINT;Tash_PEST										**1**		
FAINT;Tash_PEST;AT_hook;FAINT;Tash_PEST;FAINT										**1**		
***Protease Domains***	***Bb***	***Ch***	***Cp***	***Pb***	***Pc***	***Pk***	***Pf***	***Pv***	***Py***	***Ta***	***Tp***	***Tg***
Rhomboid	**8**	**2**	**3**	**7**	**5**	**8**	**8**	**8**	**7**	**5**	**3**	**5**
Peptidase_S8	**1**		**2**	**4**	**2**	**3**	**3**	**3**	**2**			**10**
Peptidase_S8;EGF_CA												**1**
**Total number**	**18**	**16**	**22**	**23**	**21**	**30**	**106**	**41**	**28**	**116**	**137**	**60**

* All Pfam domains found in any candidate protein from any of these apicomplexan parasites are shown. Species: *Bb*, *Babesia bovis*; *Ch*, *Cryptosporidium hominis*; *Cp*, *Cryptosporidium parvum*; *Pb*, *Plasmodium berghei*; *Pc*, *Plasmodium chabaudi*; *Pk*, *Plasmodium knowlesi*; *Pf*, *Plasmodium falciparum*; *Pv*, *Plasmodium vivax*; *Py*, *Plasmodium yoelii*; *Ta*, *Theileria annulata*; *Tp*, *Theileria parva*; *Tg*, *Toxoplasma gondii*.

Prediction of secretory signal peptides and signal anchor sequences were performed using SignalP (http://www.cbs.dtu.dk/services/SignalP/) [45], and transmembrane domain identification was conducted using TMHMM (http://www.cbs.dtu.dk/services/TMHMM/) [46] applying default parameters.

### Data sources and data comparison

Parasite genomic and proteomic datasets were obtained from the source genome databases: *T. gondii* from ToxoDB.org v4.1; *Plasmodium* spp. from PlasmoDB.org v5.3; *Cryptosporidium* spp. from CryptoDB.org v3.5; *T. parva* from ftp://ftp.tigr.org/pub/data/Eukaryotic_Projects/t_parva/annotation_dbs/; *T. annulata* from ftp://ftp.sanger.ac.uk/pub/pathogens/T_annulata/TANN.GeneDB.pep. Protein sequences for *B. bovis* were obtained by searching the reference sequence collection of GenBank for species name ‘*Babesia bovis*’. Human protein sequences were obtained from http://www.ensembl.org, v46 (ncbi36)(ftp://ftp.ensembl.org/pub/release46/homo_sapiens_46_36h/data/fasta/pep/Homo_sapiens.NCBI36.46.pep.all.fa.gz). When necessary, published protein sequences were mapped to the current genome sequences used in this study.

### Projection-based identification of the parasite-host interactome

Two strategies (designated *Phifam* and *Phint*) were explored to identify host cell proteins that may interact with parasite proteins containing one or more Pfam domains in known microneme proteins. First, *T. gondii* and *P. falciparum* proteins from Table 1 were filtered to include only those with a predicted signal sequence or signal anchor motif to enhance the specificity of possible parasite interacting domains. Excluding microneme protease domains, these proteins contain ten adhesive Pfam domains likely to be present on the surface of the parasite, where they could potentially interact with the host cell: AMA-1 (apical membrane antigen), Duffy_binding, Merozoite_SPAM, EGF (epidermal growth factor), EGF_2, EGF_CA, MSP1_C, PAN_1, TSP_1, VWA.

The *Phifam* (parasite-host-iPfam) dataset was constructed by identifying potential partners based on domain-domain interactions known from the PDB structural database. Pfam domains associated with the adhesive domains noted above were extracted from the iPfam [47] database (ftp://ftp.sanger.ac.uk/pub/databases/Pfam/releases/Pfam20.0/database_files/interactions.txt.gz) if (and only if) PDB structure(s) include the two interacting domains in different proteins. Ten interacting Pfam domains were identified: Binary_toxB, FG-GAP, ICAM_N, Integrin_alpha2, Lectin_C, LRR_1, LRRNT, Trypsin, Recep_L_domain, Tissue_fac. These domains (known to interact with ten adhesive Pfam domains noted above) were then used to search the predicted human proteome.

The *Phint* (parasite-host-interactome) dataset is based on protein-protein interactions known from a variety of *in vitro* and *in vivo* genomic-scale experimental studies, such as yeast 2-hybrid screens. First, all human proteins containing at least one of the ten adhesive domains noted above were identified. Candidate interacting proteins were then identified based on experimentally validated interaction annotations in the Human Protein Reference Database (HPRD, [48]) (http://www.hprd.org).

For evaluating performance, a dataset of host proteins known to interact with *P. falciparum* and *T. gondii* (Supplemental Table S18) was collected through extensive manual review of available literature databases.

### Molecular and cell biological techniques

Candidate genes were amplified from a *T. gondii* cDNA library using gene-specific oligonucleotide primers (see supplemental methods for the sequences of primers employed for genes 8.m00176, 8.m00177, 8.m00178, 8.m00179, 76.m01642, 44.m04666, 80.m00085 and 145.m00588) and subcloned into the *Avr*II/*Bgl*II sites in *p*tub-YFP/sagCAT [15]. For lower expression and HA (hemagglutinin) epitope tagging, genes were cloned into *p*min-HA [49]. The identity and sequence fidelity of all subclones was confirmed by sequencing.

Transfections were performed as previously described [50] and transfected parasites seeded into 6-well plates containing cover slips to facilitate sample processing and microscopic observation. Immunofluorescence assays were performed as previously described [51] using primary and secondary antibodies as noted below, and samples were visualized using a Leica DM IRBE inverted microscope equipped with a 100W Hg-vapor lamp, motorized filter wheel, and an Orca-ER digital camera (Hamamatsu). Image acquisition and manipulation were carried out using OpenLab software (Improvision). Primary antibodies used in this study included a rabbit polyclonal anti-*Tg*MIC10 (kindly provided by Dr. Vern Carruthers; 1∶10,000), mouse monoclonal anti-*Tg*ROP2/3/4 ([52]; 1∶10,000) and rat polyclonal anti-HA (Roche; 1∶1,000). Secondary antibodies: anti-rabbit Alexa 594, anti-mouse Alexa 488 and anti-rat Marina blue (all from Molecular Probes; 1∶3,000).

## Results

### Mining apicomplexan parasite genomes for candidate proteins associated with apical organelles

Most known microneme proteins contain functional domains (including adhesive and protease domains) thought to be involved in parasites attachment to– and invasion of –their host cells [26], [38], [53]. In order to define a more comprehensive list of domain patterns that may be relevant to apicomplexan pathogenesis, we collected the sequences of all known microneme proteins from the UniProt [54], GenBank and PubMed literature databases [55]. 55 proteins were collected from 8 species: *Babesia bovis*, *Cryptosporidium parvum*, *Eimeria tenella*, *Neospora caninum*, *Plasmodium falciparum*, *Sarcocystis muris*, *Theileria parva*, and *Toxoplasma gondii* (Fig. 1). These sequences were then searched against the Pfam database [44], identifying a total of 12 functional domains (Fig. 1). These domains are of various lengths, and may occur in isolation or in combination with others.

Apart from two protease domains (Rhomboid, Peptidase_S8) and two domains of unknown function (FAINT (frequently associated in *Theileria*), GETHR), the remaining 8 domains are typically found in proteins known to mediate adhesion to, or interaction with, other proteins or carbohydrate moieties. Some domains, such as AMA-1 (apical membrane antigen) and FAINT (frequently associated in *Theileria*) are exclusive to apicomplexan parasites, and may therefore be useful as diagnostic or therapeutic targets. PAN domains are specific to coccidia (*Toxoplasma*, *Neospora*, *Eimeria*, *Sarcocystis*), while the Duffy-binding domain is present only in *Plasmodium* species. Domains such as PAN, TSP-1, VWA and EGF (epidermal growth factor superfamily, including EGF, EGF-CA&EGF-2) are usually present as tandem copies, sometimes in combination with other adhesive domains. For example, the TRAP family of proteins present in multiple species contains a combination of VWA and TSP-1 domains (line 2 in Fig. 1 and [26]).

In order to identify additional proteins with similar or novel domain patterns, all available completed apicomplexan genome sequences were searched for proteins predicted to contain at least one of the domains cited above (see Methods). Twelve apicomplexan genomes (*B. bovis*, *C. hominis*, *C. parvum*, *P. falciparum*, *P. vivax*, *P. knowlesi*, *P. yoelii*, *P. berghei*, *P. chabaudi*, *T. annulata*, *T. parva*, and *T. gondii*) yielded a total of 618 proteins, with *C. hominis* harboring the fewest (16 proteins) and *T. parva* the most (137 proteins) (See Table 1 and Supplemental Tables S1, S2, S3, S4, S5, S6, S7, S8, S9, S10, S11, S12.) Although this dataset was constructed without explicit reference to secretion, 310 of the proteins identified (∼50%) are predicted to contain a signal peptide (275) or signal anchor (35). This represents a 2.5-fold enrichment of secretory proteins relative to the entire proteome, validating the strategy for enriching the predicted secretome.

The distribution of proteins containing functional domains associated with adhesion across different species shows several interesting patterns. TSP-1, AMA-1, VWA, VWA+TSP-1 and EGF-like domains are found in most species, suggesting that they play a common functional role during the invasion process. Species-specific expansions of domain patterns are also noticeable, such as the >100 FAINT domain-containing proteins in *Theileria* [56]. *P. falciparum* is also known to exhibit an expansion of Duffy-binding domains [57], which are much less common in other *Plasmodium spp.* and restricted to this genus [58]. *Toxoplasma* displays an expansion of PAN, TSP-1 and EGF domain-containing proteins, suggesting a role for these adhesive domains in *T. gondii* interaction with the host. Only a single instance of the *Tg*MIC2 adhesive domain pattern (VWA+tandem TSP-1) was identified in *T. gondii* (Table 1), which may explain the inability to knock out this gene unless the *E. tenella* ortholog (*Et*MIC1) is provided in *trans* [59].

This analysis also identifies several domain patterns not previously described. For example, proteins with adhesive domain patterns from known microneme proteins in other apicomplexan species were discovered in *T. gondii*, such as a VWA domain-containing protein and eight TSP-1 domain-containing proteins (Table 1). Novel adhesive domain patterns include a combination of TSP-1 with Notch (implicated in developmental signaling [60], 61) in *T. gondii* and *C. parvum* (Table 1, lines 8&9). The *T. gondii* protein also includes a Sushi domain, which is often associated with adhesive domains in metazoa [62].

As expected, protease domains were identified in most species examined. Among the many cellular activities in which proteases are involved, proteolysis is known to be essential for the maturation of several microneme proteins [37], [40], [53], [63]–[65]. Rhomboid family proteases are found in all apicomplexan parasites (Table 1), highlighting their importance in microneme protein processing. (One of the six previously reported rhomboid proteases in *T. gondii* [63], *Tg*ROM6, is absent from this candidate list, due to absence of the corresponding gene model in the protein dataset employed for this study.) No peptidase_S8 domains were identified in *Theileria*, although this may be attributable to incomplete or inaccurate gene models, as is almost certainly the case for *C. hominis*.

### Comparison of computationally derived data with available apicomplexan proteomic datasets

In order to further characterize candidate proteins identified *in silico*, we compared these predictions with several proteomics datasets intended to identify secreted or apical proteins in apicomplexan parasites (Table 2, additional details in Supplemental Tables S13 and S14). Proteomics data from *T. gondii* [19]–[21], [66] and *Plasmodium spp.* [10], [12], [67], [68] exhibit little overlap with each other or with the computationally-defined dataset described above, probably because none of these datasets comes close to saturation sampling of the secretome, and each was motivated by different experimental rationales and employed different protocols. The highest degree of overlap between the dataset defined by this study and published proteomics datasets [19] includes six known microneme proteins and four members of the expanded family of PAN domain-containing proteins noted above. A TSP domain-containing protein identified in our analysis was also present in the calcium dependent secretome proteomics dataset [21]. Overall, proteomics datasets contained very few of the adhesive domain-containing proteins identified computationally, suggesting that these proteins are either not expressed, not enriched in other secretory organellar fractions, or not readily detected by current proteomics methodology. As many microneme and erythrocyte surface proteins are known to contain adhesive domains [69], it appears likely that the computational dataset described above provides a useful complement to proteomic studies.

**Table 2:** Comparison of computational and proteomics datasets for the secretomes of *T. gondii* and *P. falciparum*.

*Toxoplasma gondii*	I	II	III	IV	V
I computational secretome (this study)	*60*	10	0	1	5
II experimental secretome [19]		*75*	7	0	13
III rhoptry proteome [20]			*48*	0	2
IV detergent-resistant proteome [66]				*179*	0
V calcium-dependent secretion [21]					*15*

*Plasmodium falciparum*	I	II	III	IV	V
I computational secretome (this study)	*106*	1	0	2	1
II Maurer's cleft proteome [12]		*50*	4	11	4
III rhoptry proteome [10]			*24*	3	2
IV detergent-resistant proteome [68]				*62*	2
V parasite-infected RBC surface [67]					*36*

### Subcellular localization of candidate adhesive domain-containing proteins

**Figure 2:**
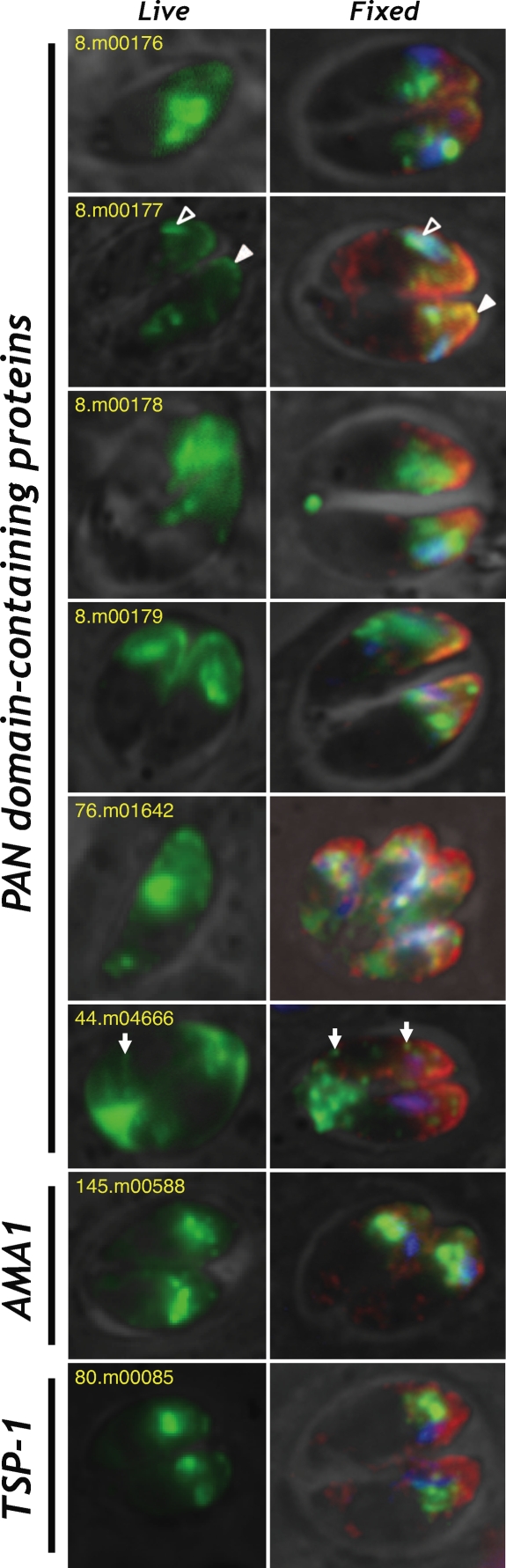
Subcellular localization of eight candidate adhesive domain-containing proteins in *T. gondii*. Left: Direct fluorescence of YFP in *T. gondii* tachyzoites expressing fusion constructs of eight candidate adhesive domain-containing proteins (numbers indicate gene IDs). Right: Fixed parasites transfected with YFP- or HA-tagged transgenes were stained with anti-HA (76.m01642 only) or anti-YFP (green), anti-*Tg*MIC10 (red), and anti-*Tg*ROP2/3/4 (blue). Open and filled arrowheads in 8.m00177 indicate rhoptry and microneme staining, respectively; arrows in 44.m04666 indicate dense granules.

*T. gondii* is the most genetically and cell biologically tractable apicomplexan parasite [50]. We therefore tested the subcellular localization of several candidate adhesive domain-containing proteins in this system. The 60 candidates identified in *T. gondii* include 9 previously validated microneme proteins, 13 with protease domains only (which were excluded from further analysis in order to focus on putative adhesins), 16 which lack a predicted signal peptide (likely due to inaccurate annotation), and 8 more for which gene models are ambiguous. Of the remaining 14 genes, 6 proved difficult to amplify or clone, but 8 were successfully engineered as C-terminal fusions with a yellow fluorescent protein (YFP) reporter. To guard against artifacts attributable to fusion with YFP or overexpression under control of the relatively strong ß-tubulin promoter [70], these genes were also fused to a hemagglutinin (HA) epitope tag rather than YFP, and expressed under control of the relatively weak minimal promoter of dihydrofolate reductase-thymidylate synthase [71], as described under Methods. Following transfection, subcellular localization was determined by direct fluorescence imaging of YFP (Fig. 2 left), or staining of fixed parasites with antibodies specific for native microneme proteins, rhoptry proteins, and either YFP or the HA epitope tag (Fig. 2, right). In all cases, *tub*-X-YFP and *dhfr*-X-HA transfectants yielded identical results (not shown).

Seven of the eight constructs tested directed YFP to the apical end of the parasite (at right in all panels of Fig. 2). For all of these genes, labelling of *Tg*MIC10 (red) and *Tg*ROP2/3/4 (blue) demonstrates at least partial colocalization with both rhoptry and microneme markers (cf. arrow heads for 8.m00177 in Fig. 2). Apparent dual localization to rhoptries and micronemes has previously been reported by others [19], [22], although in all cases (including this report) it is unclear whether the native protein is targeted to both organelles (alternatively, this could be a consequence of inefficient targeting due to recombinant engineering of fusion proteins under the control of heterologous promoters [51]). Partial colocalization with micronemes and/or rhoptries was also observed for 145.m00588 and 80.m00085 (Fig. 2, lower two panels). This may be attributable to inefficient trafficking through the secretory pathway (perhaps as a consequence of expression timing and/or recombinant protein fusion), protein-protein associations, unusual subcellular targeting signals, or other factors. Sub-apical staining reminiscent of Golgi localization was occasionally observed, as expected for proteins traversing this organelle during secretion [72].

One protein (44.m04666) was secreted into the parasitophorous vacuole, probably via dense granules (arrows), without obvious staining of the apical end. It is interesting to note that the PAN domain structure exhibited by this protein is very similar to the five other PAN domain-containing proteins tested, all of which targeted to the rhoptries and micronemes. Preliminary truncation experiments map sequences responsible for the difference in subcellular localization to a region between the signal peptide and the first PAN domain (see Supplemental Text S1, Figs. S1 and S2), indicating that organellar targeting information is not contained within the PAN domains themselves.

### *In silico* prediction of interactions between parasite and host proteins

**Figure 3:**
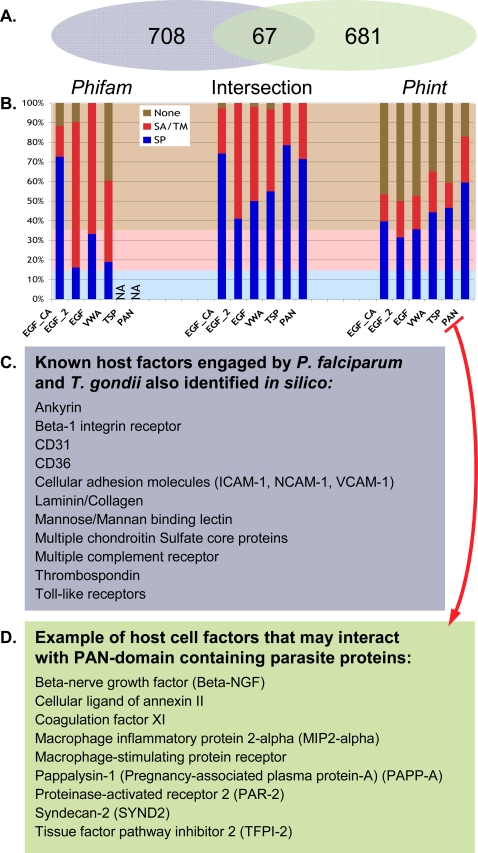
Interactomic predictions of human host partners for parasite adhesive domain-containing proteins. A: Venn diagram depicting parasite-host iPfam (*Phifam*) and the parasite-host interactome (*Phint*) datasets (see Supplemental Table S15 for complete list). B: Graphical representation of the percentage of secretory signal peptide (SP) and signal anchor (SA)/transmembrane (TM) containing proteins within *Phifam*, *Phint* and their intersection, demonstrating SP/SA/TM enrichment in the dataset of predicted interacting partners (compare with the entire human proteome, as indicated by background color). C: List of host factors known to interact with *T. gondii* and/or *P. falciparum* found in either the *Phifam* or *Phint* datasets (Supplemental Table S18). D: A selected list of host proteins predicted to interact with PAN domain-containing proteins.

Adhesive domain proteins associated with the micronemes and/or rhoptries are likely to be secreted, and since many of the adhesive domains present in our dataset are conserved across life –including in organisms for which genomic-scale interactome datasets are available– we devised a computational approach using available domain-domain and human protein-protein interaction datasets to predict putative parasite-host interactions (see Methods). This analysis yielded two sets of predictions: *Phifam* (for parasite-human-iPfam), containing 775 candidate interacting partners, and *Phint* (for parasite-human interactome), containing 748 candidates; 67 human proteins are included in both datasets (Fig. 3A and Supplemental Table S15).

Although there is undoubtedly a great deal of noise in these datasets, two observations suggest enrichment in proteins of interest. First, both datasets are enriched in secretory signal sequences, signal anchors and transmembrane domains (71% *Phifam* and 56% *Phint*, vs. 35% in the entire human genome) (Fig. 3B). Second, these datasets are highly enriched in host proteins known to be involved in parasite-human interactions, including chondroitin sulfate proteoglycan core proteins (CSPCP) [73], [74], toll-like receptors [75], mannose binding lectin [76], CD36 [77], [78], CD31 [79] and interacellular adhesion molecule 1 (ICAM-1) [80], [81] (Fig. 3C). The combined dataset successfully identifies (without training) thirteen of the eighteen (72%) human proteins known to interact with *P. falciparum* or *T. gondii* based on an extensive review of the literature (Supplemental Table S18).

Fig. 3D (and Supplemental Table S15) highlights host proteins, including several surface receptors, that are likely to interact with PAN domain-containing parasite proteins. As noted above, PAN domain proteins constitute an expanded family in *T. gondii*, including microneme proteins thought to be involved in host-cell attachment, but no host cell partners have yet been identified. Further analysis, such as filtering based on tissue-specific expression, yields host cell factors that may play a role in tissue tropism (Supplemental Table S15).

## Discussion

This study aims to merge *in vivo* and *in silico* methods to identify parasite and host proteins likely to be involved in parasite infection of human cells. The first step was to computationally identify candidate apicomplexan adhesive domain-containing proteins, utilizing Pfam domain patterns present in known microneme proteins (Fig. 1). Such adhesive domains are known to be found in microneme proteins, and are likely to be involved in host-parasite interactions. This approach yielded a total of 618 proteins from twelve apicomplexan parasite genomes (Table 1). Although this dataset imposed no explicit selection for signal sequences (or transmembrane domains), the resulting list is highly enriched in signal-sequence-containing proteins, arguing that it does indeed provide insight into the parasite secretome. Many of those proteins without obvious signal sequences are probably inaccurately annotated, as first exon prediction is notoriously difficult. For example, *T. gondii* gene model 55.m00005, corresponding to AMA1, lacks a signal peptide, in contrast to the experimentally determined sequence (Genbank # AAB65410). Because sensitivity is of primary importance for identifying candidate microneme proteins for validation in the laboratory, the predicted parasite proteomes were not filtered to require signal sequences (SP+ gene models are flagged in the accompanying tables, however).

Computational predictions were tested by cell biological validation of candidate proteins in *T. gondii*, resulting in colocalization of all but one of the eight candidates tested with rhoptries and micronemes in the parasite's apical end (Fig. 2). The value of this computationally derived dataset becomes apparent by comparison with proteomics studies on the parasite invasion complex and secreted organelles (Table 2): based on experimental validation, the low degree of overlap suggests complementarity of incomplete datasets, rather than poor quality data. In addition, using the secretome-enriched dataset of candidate parasite adhesins, we exploited human interactome datasets to identify possible host-cell partners. Future studies will be required to test these predictions in the lab.

Homology-based BLAST methods have previously been used to identify possible microneme proteins in apicomplexan parasites [82]. In contrast to pairwise similarity search methods, the profile-based HMM models employed by Pfam [44] construct libraries of evolutionary conserved domains across a range of species, and are therefore able to detect remote homologies in new species [83], [84]. Pfam is particularly useful for analyzing multi-domain proteins. Our analysis also enables the grouping of proteins from different apicomplexan parasites into families, based on their domain patterns (Table 1).

Since this analysis is limited to previously-defined domains included in the Pfam database, it is unable to detect microneme proteins such as *Tg*MIC5 or *Tg*MIC10 (Genbank CAA70921, AAG32024), which lack such domains, or *Tg*MIC1, for which the MAR domain [85] has only recently been described. Efforts to apply motif-recognition algorithms (MEME [86], TEIRESIAS [87]) to these proteins failed to yield statistically significant results. We are also likely to miss proteins with degenerate adhesive domains exhibiting weak similarity to established HMM profiles, such as the TSP-1 domains in *Pf*MTRAP [82], *Bb*TRAP [88], and *Cp*TRAP-C1 [89], and the Sushi domain in *Pf*ASP [90]. Detecting such domains would require case-specific cut-offs, which were not employed to avoid introducing a degree of subjectivity and likely false positive results when applied on a genome-wide scale. Because they are based on primary sequence only, Pfam also fails to detect structural motifs such as domain I and II of *Pv*AMA1, which share structural similarity with PAN domains [91], but lack significant primary sequence conservation with the Pfam HMM profile for PAN domains. Note, however, that putative AMA1 proteins were nevertheless identified in this study, based on the HMM profile for AMA1. As additional apicomplexan-specific domains are identified and incorporated into Pfam, and as the accuracy of genome annotation for these species improves [92], the analysis pipeline described above can be easily updated to facilitate the identification of additional candidate proteins. It may also become possible to apply similar approaches to rhoptry proteins, which are noticeably lacking in Pfam domains identified to date.

One might consider expanding the identification of microneme proteins through an iterative approach, using domains (see Table 1) associated with the original adhesive domains (see Fig. 1) to seed a new search. Unfortunately, many of adhesive domain-containing proteins also harbor highly abundant motifs (e.g. zf-C3HC4, Myb_DNA-binding, AT-hook), resulting in a very high false positive rate upon iteration of a purely computational domain recognition strategy. However, biological confirmation should permit iteration using a larger set of validated microneme proteins.

Adhesive molecules play important roles in ligand binding, cell-cell and cell-extracellular matrix interactions in higher eukaryotes [28], [29]. In apicomplexan parasites, these molecules have similar functions, albeit for the purpose of invasion [35], [36], [93]. Identification of many Pfam adhesive domains in known microneme proteins suggests functional conservation across apicomplexan parasites and higher eukaryotes, and indicates a likely common ancestry. Genome-wide identification of known microneme Pfam domains in multiple apicomplexan species also reveals species-specific expansions (PAN, TSP-1 and EGF, Duffy-binding domains; Table 1). In contrast to the expansion of Duffy-binding domains in *P. falciparum*, the expansion of PAN and EGF domains appears to be coccidian specific. Expansion of particular families is suggestive of species-specific functions, perhaps related to the type of cells that they invade. It is tempting to speculate that the expansion of PAN domain-containing proteins in coccidians correlates with their unique cellular tropisms, such as the broad host range of *T. gondii*. Indeed, several PAN domain-containing microneme proteins have been identified [35], [94]–[97], and *Tg*MIC4 has been shown to bind to host cells [35]. Parasites also harbour adhesive proteins not associated with micronemes, but surprisingly few have been described to date, and most of these play important roles in cell attachment and invasion (*Pf*EMP1 proteins [98] contain Duffy domains, and several *Pf*MSP proteins [99]–[102] contain EGF domains).

One of the PAN domain-containing proteins tested (44.m04666) did not localize to the apical end of *T. gondii* and was secreted into the parasitophorous vacuole probably via dense granules. To our knowledge, this is the first description of a dense granule protein containing adhesive domains. Sequence comparison of the PAN domains in *T. gondii* reveals that some represent gene duplications while others contain domain duplications (unpublished observations).

Transient transfection studies using epitope- and/or YFP-tagged transgenes reveal that all of eight proteins tested in this study targeted to the secretory organelles, and all but one to the specialized organelles at the apical end of the parasite (Fig. 2). It may be significant that we were unable to produce stable transgenics for any of the genes examined in this study, perhaps suggesting that the abundance and/or native structure of proteins they encode may be critical for parasite survival.

Several of the recombinant proteins tested in this study appear to target to both micronemes and rhoptries. It is possible that such dual localization [19], [22] is a consequence of inefficient trafficking of recombinant fusion proteins, as previously suggested [51]. It seems unlikely that this pattern could be attributable to aberrant transcription, however, as similar results were observed using both weak (*dhfr-ts*) and strong (ß-tubulin) promoters, and proper targeting has previously been observed for validated microneme and rhoptry proteins under the control of these (and other) promoters [15]. This contrasts with *Plasmodium*, where the ∼48 hr mitotic cycle and profound subcellular reorganization distinguishing rings, trophozoites and schizonts makes transcriptional control an important mechanism for regulating subcellular distribution (*Toxoplasma* maintains all organelles throughout its ∼7 hr mitotic cycle [49]).

Microneme/rhoptry localization is clearly distinct from targeting to other destination organelles in the secretory pathway, however, such as dense granules (cf. 44.m04666) and the apicoplast [51]. Five candidate microneme proteins identified in our analysis overlap with candidate apicoplast proteins from the first-draft annotation of the *P. falciparum* genome, but all of these appear to be false-positives in the apicoplast dataset: *Pf*CSP (PFC0210c) [103] and *Pf*TRAMP (PFL0870w) [104] are known to be secreted by apical complex organelles, and by homology to other microneme proteins, this is likely true for thrombospondin-related sporozoite protein (TRSP) [105] homolog PFA0200w and the rhomboid protease PF13_0312; no information is available on localization for MAL8P1.45, but this hypothetical protein lacks any obvious plastid targeting signal or phylogenetic affinity to known plastid proteins.

The adhesive domains used in this study are both structurally and functionally conserved across life. We took advantage of this feature to design an *in silico* approach for the identification of host cell proteins that may interact with candidate microneme proteins. For proof of principle, the candidate microneme list was restricted to SP+ proteins, in order to maximize specificity. Using two interaction databases (iPfam and the human interaction database), a total of 1456 possible human interacting partners were identified. The intersection between *Phifam* (iPfam based) and *Phint* (human interactome based) datasets showed only 67 proteins common to both. This low number is explained by the nature of the data present in iPfam and the human interactome. For example, because PAN domains and TSP domains have never been found to interact with other domains on different proteins in the iPfam database, proteins containing only PAN or TSP domains are excluded from the intersection. Conversely, the human interactome dataset includes interaction partners that are not necessarily based on domain-domain interactions, eliminating from the intersection any interactions outside of non-Pfam domains. It will therefore be important to explore each dataset independently, although the intersection may be considered as the most likely short list of human interacting partner proteins. Of note, both *Phifam* and *Phint* were enriched in proteins containing secretory signal sequences, signal anchors, and transmembrane domains (Fig 3B), suggesting that this dataset is enriched in host proteins that reside on the surface of cells, where they could potentially interact with parasites. Although the lack of a validated test set makes it difficult to comprehensively assess the validity of these predictions, most of the host proteins that have previously been shown to interact with parasites are represented in this dataset (Fig. 3C).

Two recent studies have employed similar computational approaches for interacting partner identification, based on predicted whole parasite vs. whole host interactions [106], [107]. Such genomic strategies complement the work presented here, in which we have restricted our analysis to parasite proteins that are likely to be involved in the invasion process based on the presence of surface adhesive domains. All of these approaches identify candidate interactions, and their intersection (Supplemental Tables S16 and S17) provides a relatively short list of parasite and host cell proteins that warrant further experimental validation.

## Supporting Information

Text S1 (0.05 MB PDF)

Figure S1 (0.01 MB PDF)

Figure S2 (0.28 MB PDF)

Table S1 List of candidate microneme proteins for *Toxoplasma gondii*.
(0.08 MB XLS)

Table S2 List of candidate microneme proteins for *Cryptosporidium hominis*.
(0.03 MB XLS)

Table S3 List of candidate microneme proteins for *Cryptosporidium parvum*.
(0.04 MB XLS)

Table S4 List of candidate microneme proteins for *Plasmodium falciparum*.
(0.24 MB XLS)

Table S5 List of candidate microneme proteins for *Plasmodium vivax*.
(0.08 MB XLS)

Table S6 List of candidate microneme proteins for *Plasmodium knowlesi*.
(0.04 MB XLS)

Table S7 List of candidate microneme proteins for *Plasmodium yoelii*.
(0.04 MB XLS)

Table S8 List of candidate microneme proteins for *Plasmodium berghei*.
(0.03 MB XLS)

Table S9 List of candidate microneme proteins for *Plasmodium chabaudi*.
(0.03 MB XLS)

Table S10 List of candidate microneme proteins for *Theileria parva*.
(0.16 MB XLS)

Table S11 List of candidate microneme proteins for *Theileria annulata*.
(0.18 MB XLS)

Table S12 List of candidate microneme proteins for *Babesia bovis*.
(0.03 MB XLS)

Table S13 Detailed results of comparisons between *T. gondii* computational data from this study and various proteomics datasets.
(0.05 MB XLS)

Table S14 Detailed results of comparisons between *P. falciparum* computational data from this study and various proteomics datasets (see Table 2).
(0.04 MB XLS)

Table S15 Complete *Phint* and *Phifam* datasets (see Fig. 3).
(1.86 MB XLS)

Table S16 Comparison of candidate human interacting partners from the *Phint* and *Phifam* datasets with those published by [106] (David *et al.* 2007) and [107] (Dyer *et al.* 2007).
(0.71 MB XLS)

Table S17 Comparison of candidate parasite interacting partners with those published by [106] (David *et al.* 2007) and [107] (Dyer *et al.* 2007).
(0.23 MB XLS)

Table S18 List of human proteins reported to be engaged by *P. falciparum* and/or *T. gondii* (including those not detected by our search methods).
(0.01 MB PDF)
