# Supplementary material for: In Silico Identification of Specialized Secretory-Organelle Proteins in Apicomplexan Parasites and In Vivo Validation in Toxoplasma gondii
Source: PLoS One. 2008 Oct 31;3(10):e3611. doi: 10.1371/journal.pone.0003611 (PMC2575384; doi:10.1371/journal.pone.0003611)
Supplement: Text S1 — (0.05 MB PDF) [file pone.0003611.s001.pdf]

Supporting information for: ***In silico* Identification of Specialized Secretory-Organelle Proteins in Apicomplexan Parasites and *In vivo* Validation in *Toxoplasma gondii***

Materials and Methods

Primers used to amplify and clone candidate *T. gondii* genes. Primers used to generate the constructs for genes 8.m00176, 8.m00177, 8.m00178, 8.m00179, 76.m01642, 44.m04666, 80.m00085 and 145.m00588 in text are as follows:

8.m01776: TgTS2357F-Bgl2 (5'-CGCAGATCTATGGACGCGTTGAAAACTTTATTGAACAAG-3')
8.m01777: TgTS2358F-Bgl2 (5'-CGCAGATCTATGGAGACTGTGACGACTCTGTTCAACCGG-3')
8.m01778: TgTS2359F-Bgl2 (5'-CGCAGATCTATGGACGCGCTGAAGACTGTGTTCAACCGG-3')
8.m01779: TgTS2361F-Bgl2 (5'-CGCAGATCTATGGACGCGCTGAAGACTGTGTTCAACCGG-3')
Since all of above four genes have exactly same C-terminus sequences, they share a universal oligo TgTS23UR-Avr2 (5'-CGCCCTAGGGCATGTGATATCGCCTGCTTTGGAGTTCGC-3')
76.m01642: 76.m01642F-Bgl2 (5'-GCGAGATCTATGCCTCACCGACGGAGTCAACCTTGGCGAA-3') and 76.m01642R-Avr2 (5'-GCGCCTAGGCGATGCAATAGCAAGGCTTCGAAAAACGAA-3')
44.m04666: TgTS5871F-Bgl2 (5'-CGCAGATCTATGGTTTCCAAGCGTGTTCAAACCGCACTA-3') and TgTS5871R-Avr2 (5'-CGCCCTAGGTATGCCACAGTTCGTGTCGCCAGAGATGTA-3')
80.m00085: 80.m00085F-Bgl2 (5'-GCGAGATCTATGGTTGTTTCCTGTCTCTGTACCGCGAT-3') and 80.m00085R-Avr2 (5'-GCGCCTAGGGAGGTAGTTGTCCCGTGTCCGTT-3')
145.m00588: 145.m00588F-Bgl2 (5'-GCGAGATCTATGACGACGCAAACAACCACGAAA-3') and 145.m00588R-Avr2 (5'-GCGCCTAGGCCTTTGTGCCATCCAGTATATCAGTCC-3')

Multiple sequence alignment of PAN domain-containing candidate proteins. Five PAN domain-containing candidate *T. gondii* proteins (8.m00176-9, 44.m04666) are aligned by using ClustalW with default parameters. Alignment was manually adjusted to highlight the region between the SP and the first PAN domain.

Analysis of targeting motif in PAN domain-containing candidate proteins of *T. gondii*. To determine if the region between the SP and the first PAN domain was required for targeting to the invasion complex we performed deletion and domain swapping experiments as follows.

Deletional analysis: To determine if the region between the SP and the first PAN domain was necessary for targeting, we deleted the PAN domains (PAN) from two PAN domain-containing proteins with different subcellular localization (8.m00177, invasion complex and 44.m04666, secreted via dense granules) (Supplemental Fig. S2).

Domain swapping: To assess the effect of the PAN domains from 8.m00177 and 44.m04666 on targeting we performed a Domain swapping experiment where the PAN domains from 8.m00177 were swapped with the PAN domains from 44.m04666. This leads to two hybrid constructs: 8.m00177PAN44, in which the PAN domains of 8.m00177 are replaced by the PAN domains of 44.m04666; 44.m04666PAN8, in which the PAN domains of 44.m04666 are replaced by the PAN domains of 8.m00177 (Supplemental Fig. S2).

Primers used to generate the various constructs were as follows:

8.m00177ΔPAN: truncation was amplified from 8.m00177 using the following primer pairs TgTS2358F-Bgl2 (5’-CGCAGATCTATGGAGACTGTGACGACTCTGTTCAACCGG-3’) and TgTS5871p1R-Avr2 (5’-CGCCCTAGGGTCATTCGGGATGTTGAGTCTCAGCAGGTC-3’).

8.m00177PAN44: The PCR product generated by amplification from 8.m00177 using the following primer pairs TgTS2358p1R-Sph1 (5’-CGCGCATGCGCTATATCCTGTCGTCTCCACCTCAGCGAA-3’) and TgTS2358F-Bgl2 (5’-CGCAGATCTATGGAGACTGTGACGACTCTGTTCAACCGG-3’) was ligated to the PCR product generated by amplification from 44.m04666 using the following primer pairs TgTS5871PAN1F-Sph1 (5’-CGCGCATGCTGCCCAGTCCAGCAAGAGCAAACAAATTTC-3’) and TgTS23UR-Avr2 (5’-CGCCCTAGGGCATGTGATATCGCCTGCTTTGGAGTTCGC-3’). The ligation product was subcloned into the Toxoplasma expression vector.

44.m04666ΔPAN: truncation was amplified from 44.m04666 using the following primer pairs TgTS5871F-Bgl2 (5’-CGCAGATCTATGGTTTCCAAGCGTGTTCAAACCGCACTA-3’) and TgTS5871p1R-Avr2 (5’-CGCCCTAGGGTCATTCGGGATGTTGAGTCTCAGCAGGTC-3’) and subcloned into the Toxoplasma expression vector.

44.m04666PAN8: The PCR product generated by amplification from 44.m04666 using the following primer pairs TgTS5871p1R-Sph1 (5’-CGCGCATGCGTCATTCGGGATGTTGAGTCTCAGCAGGTC-3’) and TgTS5871F-Bgl2 (5’-CGCAGATCTATGGTTTCCAAGCGTGTTCAAACCGCACTA-3’) was ligated to the PCR product generated by amplification from 8.m0017 using the following primer pairs TgTS2358PAN1F-Sph1 (5’-CGCGCATGCTGCCTTGAAAAAGACAAGGAGTACGTTGGT-3’) and TgTS23UR-Avr2 (5’-CGCCCTAGGGCATGTGATATCGCCTGCTTTGGAGTTCGC-3’). The ligation product was subcloned into the Toxoplasma expression vector.

Results

Identification of a region responsible for targeting proteins to apical end in *T. gondii*

While conducting multiple sequence alignment of PAN domain-containing proteins (Supplemental Fig. S1), we noticed a region that was unique to one of the PAN domain-containing proteins that was secreted (Fig. 2 of main paper and Supplemental Fig. S2). In Supplemental Fig. S1, the signal peptide (SP) of 5 PAN domain-containing proteins is highlighted in blue, the region between the SP and the first PAN domain of apically targeted proteins is indicated in purple (region A), while the same region in the secreted PAN domain-containing protein is highlighted in green (region B) and the PAN domain region is indicated in orange. While the PAN domains are fairly conserved between all of the aligned proteins, regions A and B are quite different.

To assess if region A and B are sufficient for targeting, we engineered several deletion and swapping constructs (Supplemental Fig. S2, see supplemental methods for details). The results showed that region A was sufficient for targeting to the apical region of *T. gondii* while region B was not (compare construct 8.m00177ΔPAN to 44.m04666ΔPAN in Supplemental Fig. S2). Moreover, PAN domain swapping experiments showed that the PAN domains of 44.m04666 did not change the targeting capacity of region A of 8.m00177. Interestingly the PAN domains of 8.m00177 when fused to region B of 44.m04666 caused YFP to become stuck in the endoplasmic reticulum, possibly due to aggregation.

Figure Legends

Figure S1. Multiple sequence alignment of five PAN domain-containing candidate proteins in *T. gondii*. Domains/Regions are color-coded as follows: Signal peptide, blue; PAN domain, orange; region A (region between SP and PAN domains in apically targeted candidate proteins, 8.m00176-9), purple; region B (region between SP and PAN domains in secreted candidate protein, 44.m04666), dark green.

Figure S2. Subcellular localization of PAN domain deletion and swapping constructs. Left: Construct names. Middle: Construct structures represented by blocks of domains/regions which are color-coded as Fig. S1 (see legend for detail). YFP (light green). PAN domains of 44.m04666 are diagonally shaded to be distinguished from PAN domains of 8.m0177. Right: Direct fluorescence of YFP in *T. gondii* tachyzoites expressing fusion constructs.

Tables

Table S1: List of candidate microneme proteins for *Toxoplasma gondii*.
Table S2: List of candidate microneme proteins for *Cryptosporidium hominis*.

Table S3: List of candidate microneme proteins for *Cryptosporidium parvum*.
Table S4: List of candidate microneme proteins for *Plasmodium falciparum*.
Table S5: List of candidate microneme proteins for *Plasmodium vivax*.
Table S6: List of candidate microneme proteins for *Plasmodium knowlesi*.
Table S7: List of candidate microneme proteins for *Plasmodium yoelii*.
Table S8: List of candidate microneme proteins for *Plasmodium berghei*.
Table S9: List of candidate microneme proteins for *Plasmodium chabaudi*.
Table S10: List of candidate microneme proteins for *Theileria parva*.
Table S11: List of candidate microneme proteins for *Theileria annulata*.
Table S12: List of candidate microneme proteins for *Babesia bovis*.
Table S13: Detailed results of comparisons between *T. gondii* computational data from this study and various proteomics datasets (see Table 2).
Table S14: Detailed results of comparisons between *P. falciparum* computational data from this study and various proteomics datasets (see Table 2).
Table S15: Complete *Phint* and *Phifam* datasets (see Fig. 3).
Table S16: Comparison of candidate human interacting partners from the *Phint* and *Phifam* datasets with those published by [106] (David *et al.* 2007) and [107] (Dyer *et al.* 2007).
Table S17: Comparison of candidate parasite interacting partners with those published by [106] (David *et al.* 2007) and [107] (Dyer *et al.* 2007).
Table S18: List of human proteins reported to be engaged by *P. falciparum* and *T. gondii* (including those not detected by our search methods).

Note: Tables S1-S2 and S15 include SignalP and TMHMM predictions, Pfam domain predictions and amino acid sequence for each candidate. Tables S13, S14, S16 and S17 include presence ('1') or absence ('0') information of proteins (rows) under each dataset (column).
