# Supplementary material for: In Silico Identification of Specialized Secretory-Organelle Proteins in Apicomplexan Parasites and In Vivo Validation in Toxoplasma gondii
Source: PLoS One. 2008 Oct 31;3(10):e3611. doi: 10.1371/journal.pone.0003611 (PMC2575384; doi:10.1371/journal.pone.0003611)
Supplement: Figure S1 — (0.01 MB PDF) [file pone.0003611.s002.pdf]

Chen *et al*,

Supplemental Fig. S1 (top)

```
 8.m00176MDALKTLLNKGITLAVVAAVLLQIRAVALQYPGLRNQVVNAHSFAEVETTGYSCLEKDK 60
 8.m00177METVTTLFNRKIALSFVVAAALLHNSAEALQSPGLRRQLVNVHSFAEVETTGYSCLEKDK 60
 8.m00178MDALKTVFNRRIALPVVVAAALLHNSAEALQSPGLRRQLVNSHSFAEVETTGYSCFEKGK 60
 8.m00179MDALKTVFNRRIALPVVVAAALLHNSAEALQSPGLRRQLVNAHSFAEVESTGYSCLEKGK 60
44.m04666MVSKRVQTALAVLLPGLLLAKSGVQRVDA--------Q-VDAEDLLRLNIPND-C-PVQQ 49
         *..: :  ....    *     .:*        *: ..: .:: .. . .:  :*.

 8.m00176EYVGFSLTEFTEVGDAGLCQQRCNQHPHCSFFTFYSNGNRCVLQSRKPSQEKNNADAVSG 120
 8.m00177EYVGFSLTEFTEVGDAGLCQQRCNQHPHCSFFTFYSNGNRCVLQSRKPSQEKNNADAVSG 120
 8.m00178EYVGFSLTEFAKVGDAALCQQRCNQHPQCGFFTFYSNENRCVLQSRKPSQENNNANAVSG 120
 8.m00179EYVGFSLTEFTEVGDAALCQQRCNQHPQCGFFTFYSNGNRCVLQSRKPSQEKNNANAVSG 120
44.m04666EQTNFQMAEFTGVPFNATCQRYCAEHPECAAFTYHADSRRCVLHSRTGNLAEKATARFSR 109
         * ..*.::**: *   . **: * :**.*. **:::: .****:**. .  :: :  .*

 8.m00176PKRCPLCLVDSFDFRGEANLHHKGAPGLNTLLACQQGCAAEPRCKGFLFEKRPRTCHFKT 180
 8.m00177PKRCPLCLVDSFDFRGEANLHHKGAPGLNTLLACQQGCAAEPRCKGFLFEKRPRTCHFKT 180
 8.m00178PKRCPLCLVDNYDFRGETNMHKSGAPGLNTLLACQQGCAAEPKCKAFLFEKRPRTCHFKT 180
 8.m00179PKRCPLCLVDSFDFRGEKNMHDNGVPGIKTVVECQMGCAAEPKCKGFLFQHKTKQCHFKT 180
44.m04666-KRCPTCLLDNFNVADTEDDYLVIA---MTVQGCASECRTR-GCNFYVYDKESKKCHFKR 164
         **** **:*.::. .  : :   .    *:  *   * :.  *: :::::..: ****

 8.m00176N-DNYLKALDPDTSYIAGPKTCT-DEHWCIMKDIGYRGTDSKETRANSAAECQQMCLNDE 238
 8.m00177N-DNYLKALDPDTSYIAGPKTCT-DEHWCIMKDIGYRGTDSKETRANSAAECQQMCLNDE 238
 8.m00178S-DNYLKSFHPDTSYIAGPKTCT-DEHWCIMKDIGYKGTDSKATKANSAAECQQMCLNDE 238
 8.m00179S-DNYLKSLHPDNEYVAGPKTCT-GEHWCIMKDIGYEAMYTRRSQTNSAEECQNKCLNDE 238
44.m04666HRDGIQSAIRPQQGLQVGFHNCRNTDSWCFAPNHTLQGETLKTTTASGPLHCSEICLNHA 224
           *.  .:: *:    .* :.*   : **:  :   ..   : : :... .*.: ***.

 8.m00176RCDFFTWQQAGKYCWFKAGASTASTKYNRAGDYSAPKHCGLPTTCVKERTKYAGETVATF 298
 8.m00177RCDFFTWQQAGKYCWFKAGASTASTKYNRAGDYSAPKHCGLPTTCVKERTKYAGETVATF 298
 8.m00178RCDFFTWQQAGKHCWFKAGASTASTKYNRAGDYSAPKHCGLPTTCVKERTKYAGETVATF 298
 8.m00179QCQYFTWQVSNKHCWLKHGPTIANSKYNRQGDHSAPKHCGLPTTCVKERTKYAGETVATF 298
44.m04666GCAAFSFDVGSKACHLKP-ASAFDHVAPQNGSFFGPPGCGLPQACETQGKRRAGDFLMSF 283
         *  *::: ..* * :*  .:  .    : *.. .*  **** :* .: .: **: : :*

 8.m00176PKSEVGTFESCQMKCWKTSKCVFMHFNNDGCTLSGINATAQTDANSKAGDITC-- 351
 8.m00177PKSEVGTFESCQMKCWKTSKCVFMHFNNDGCTLSGINATAQTDANSKAGDITC-- 351
 8.m00178PKSEVGTFESCQMKCWKTSKCVFMHFNNDGCTLSGINATAQTDANSKAGDITC-- 351
 8.m00179PKSEVGTFESCQMKCWKTSKCVFMHFNNDGCTLSGINATAQTDANSKAGDITC-- 351
44.m04666SPNSIKTNKGCQRRCQENHRCFHYSFGPEGCFLHGLGSVAEDVEGYISGDTNCGI 338
         . ..: * :.** :* :. :*..  *. :** * *:.:.*:   .  :** .*
```
