# Supplementary material for: In Silico Identification of Specialized Secretory-Organelle Proteins in Apicomplexan Parasites and In Vivo Validation in Toxoplasma gondii
Source: PLoS One. 2008 Oct 31;3(10):e3611. doi: 10.1371/journal.pone.0003611 (PMC2575384; doi:10.1371/journal.pone.0003611)

Chen *et al*, Supplemental Fig. S2 (top)

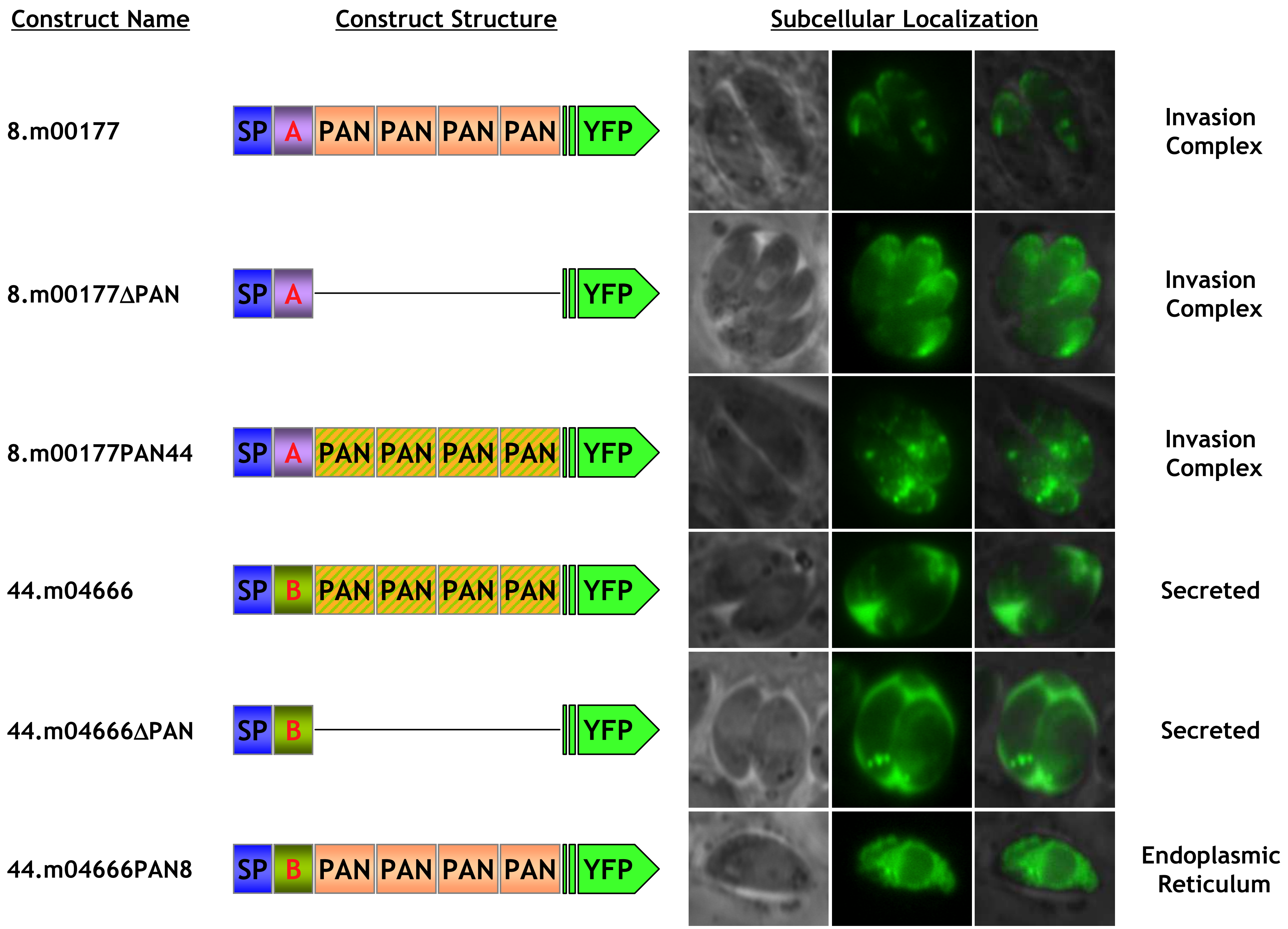

Supplement: Figure S2 — (0.28 MB PDF) [file pone.0003611.s003.pdf]
