# Supplementary material for: In Silico Identification of Specialized Secretory-Organelle Proteins in Apicomplexan Parasites and In Vivo Validation in Toxoplasma gondii
Source: PLoS One. 2008 Oct 31;3(10):e3611. doi: 10.1371/journal.pone.0003611 (PMC2575384; doi:10.1371/journal.pone.0003611)
Supplement: Table S18 — List of human proteins reported to be engaged by P. falciparum and/or T. gondii (including those not detected by our search methods). (0.01 MB PDF) [file pone.0003611.s021.pdf]

Table S 18
List of human proteins reported to be engaged by *P. falciparum* and/or *T. gondii*

Host cell receptor	Parasite Species	Parasite Ligand (if known)	Citation	In this Study	
				Phifam	Phint
Ankyrin	*P. falciparum*	PfHRP1	(1)	-	+
Band3	*P. falciparum*	MSP-1 and -9	(2)	-	-
Band 4.1	*P. falciparum*	EBA 181, MESA (PfEMP2)	(3-6)	-	-
β-1 integrin receptor	*T. gondii*		(7)	-	+
CD31/PECAM-1 (platelet/endothelial cell adhesion molecule 1)	*P. falciparum*	PfEMP1	(8,9)	-	+
CD36 (platelet glycoprotein IV)	*P. falciparum*	PfEMP1	(9-11)	-	+
CCR5	*T. gondii*	Cyclophilin	(12)	-	-
Chondroitin sulfate (CSA)	*P. falciparum*	PfEMP1	(13,14)	+	+
Complement receptor	*P. falciparum*	PfEMP1	(15)	+	+
Glycophorin A	*P. falciparum*	EBA-175	(16,17)	-	-
Glycophorin C	*P. falciparum*	EBA 140 (BABEL, EBP2)	(18)	-	-
Intracellular adhesion molecule (ICAM-1)	*Plasmodium* spp. *T. gondii*	PfEMP1 TgMic2	(11,19,20)	+	+
Laminin/collagen	*T. gondii*		(7,21,22)	-	+
Mannose/mannan binding lectin	*P. falciparum*		(23)	+	+
Neural cell adhesion molecule (NCAM-1)	*P. falciparum*		(24)	-	+
Thrombospondin	*P. falciparum*		(25)	-	+
Toll-like receptors (TLR)	*T. gondii*		(26)	+	-
Vascular cell adhesion molecule (VCAM-1)	*P. falciparum*		(20)	-	+

1. Magowan, C., Nunomura, W., Waller, K. L., Yeung, J., Liang, J., Van Dort, H., Low, P. S., Coppel, R. L., and Mohandas, N. (2000) *Biochim Biophys Acta* **1502**, 461-470
2. Li, X., Chen, H., Oo, T. H., Daly, T. M., Bergman, L. W., Liu, S. C., Chishti, A. H., and Oh, S. S. (2004) *J Biol Chem* **279**, 5765-5771
3. Lanzillotti, R., and Coetzer, T. L. (2006) *Malar J* **5**, 100
4. Lauterbach, S. B., Lanzillotti, R., and Coetzer, T. L. (2003) *Malar J* **2**, 47
5. Lustigman, S., Anders, R. F., Brown, G. V., and Coppel, R. L. (1990) *Mol Biochem Parasitol* **38**, 261-270
6. Waller, K. L., Nunomura, W., An, X., Cooke, B. M., Mohandas, N., and Coppel, R. L. (2003) *Blood* **102**, 1911-1914
7. Furtado, G. C., Cao, Y., and Joiner, K. A. (1992) *Infect Immun* **60**, 4925-4931
8. Treutiger, C. J., Heddini, A., Fernandez, V., Muller, W. A., and Wahlgren, M. (1997) *Nat Med* **3**, 1405-1408

9. Chen, Q., Heddini, A., Barragan, A., Fernandez, V., Pearce, S. F., and Wahlgren, M. (2000) *J Exp Med* **192**, 1-10
10. Ockenhouse, C. F., Tandon, N. N., Magowan, C., Jamieson, G. A., and Chulay, J. D. (1989) *Science* **243**, 1469-1471
11. Berendt, A. R., Simmons, D. L., Tansey, J., Newbold, C. I., and Marsh, K. (1989) *Nature* **341**, 57-59
12. Aliberti, J., Valenzuela, J. G., Carruthers, V. B., Hieny, S., Andersen, J., Charest, H., Reis e Sousa, C., Fairlamb, A., Ribeiro, J. M., and Sher, A. (2003) *Nat Immunol* **4**, 485-490
13. Fried, M., and Duffy, P. E. (1996) *Science* **272**, 1502-1504
14. Buffet, P. A., Gamain, B., Scheidig, C., Baruch, D., Smith, J. D., Hernandez-Rivas, R., Pouvelle, B., Oishi, S., Fujii, N., Fusai, T., Parzy, D., Miller, L. H., Gysin, J., and Scherf, A. (1999) *Proc Natl Acad Sci U S A* **96**, 12743-12748
15. Rowe, J. A., Moulds, J. M., Newbold, C. I., and Miller, L. H. (1997) *Nature* **388**, 292-295
16. Camus, D., and Hadley, T. J. (1985) *Science* **230**, 553-556
17. Sim, B. K., Chitnis, C. E., Wasniowska, K., Hadley, T. J., and Miller, L. H. (1994) *Science* **264**, 1941-1944
18. Lobo, C. A., Rodriguez, M., Reid, M., and Lustigman, S. (2003) *Blood* **101**, 4628-4631
19. Barragan, A., Brossier, F., and Sibley, L. D. (2005) *Cell Microbiol* **7**, 561-568
20. Newbold, C., Warn, P., Black, G., Berendt, A., Craig, A., Snow, B., Msobo, M., Peshu, N., and Marsh, K. (1997) *Am J Trop Med Hyg* **57**, 389-398
21. Adini, A., and Warburg, A. (1999) *Parasitology* **119 (Pt 4)**, 331-336
22. Mahairaki, V., Voyatzi, T., Siden-Kiamos, I., and Louis, C. (2005) *Mol Biochem Parasitol* **140**, 119-121
23. Klabunde, J., Uhlemann, A. C., Tebo, A. E., Kimmel, J., Schwarz, R. T., Kremsner, P. G., and Kun, J. F. (2002) *Parasitol Res* **88**, 113-117
24. Pouvelle, B., Matarazzo, V., Jurzynski, C., Nemeth, J., Ramharter, M., Rougon, G., and Gysin, J. (2007) *Infect Immun* **75**, 3516-3522
25. Roberts, D. D., Sherwood, J. A., Spitalnik, S. L., Panton, L. J., Howard, R. J., Dixit, V. M., Frazier, W. A., Miller, L. H., and Ginsburg, V. (1985) *Nature* **318**, 64-66
26. Yarovinsky, F., and Sher, A. (2006) *Int J Parasitol* **36**, 255-259
